# Erichsen on American Surgery

**Published:** 1875-01

**Authors:** 


Mr. Erichsen on American Surgery.—On November 9th, Mr. Erichsen delivered an address in London on his impressions of American surgery during his late tour in this country. It is in general favorable, as may be judged from the following extract:

Surgery in the United States certainly stands at a very high level of excellence. The hospital surgeons throughout the country have struck me as being alike practical, progressive and learned in a very high degree. In practical skill, and aptitude for mechanical appliances of all kinds, they are certainly excelled by no class ef practitioners in any country. They are thoroughly up to modern surgery in its most progressive forms, and I have never met with any class of men who are so well read and so perfectly acquainted with all that is done in their profession outside their own country. It would be a great injustice to American surgeons for it to be supposed that surgical skill is confined to the large cities, or to the few. On the contrary, I know no country in which, so far as it is possible to judge from contemporary medical literature, there is so widely diffused a high standard of operative skill, as in the country districts and more remote provinces of the United States. The bent of the mind of the American surgeon is, like ours, practical rather than scientific; in fact, there are the same mental characteristics displayed in him that we find here; the same self-reliance, the same practical aptitude, the same *curative* instinct, which leads him to consider his patient rather as a human being to be rescued from the effects of disease or injury, than as a scientific object to be studied for the advance of professional knowledge. How, indeed, can it be otherwise than that there should be such a resemblance?
—*Medical and Surgical Reporter.*

